# Global research landscape and trends of metabolomics in diabetic kidney disease: focus on immunometabolic interactions

**DOI:** 10.3389/fcdhc.2026.1791774

**Published:** 2026-07-15

**Authors:** Yuting Li, Changlin Li, Jiamin Duan, Qingli Yang, Jing Zhang, Duo Yu, Yuwei Chen, Jianing Ni, Xiaomeng Lin, Xudong Cai

**Affiliations:** Ningbo Municipal Hospital of Traditional Chinese Medicine (TCM), Affiliated Hospital of Zhejiang Chinese Medical University, Ningbo, Zhejiang, China

**Keywords:** citespace, diabetic kidney disease, inflammation, metabolomics, pubmed, web of science

## Abstract

**Introduction:**

Diabetic kidney disease (DKD), a devastating microvascular complication of diabetes mellitus, arises from intricate crosstalk between metabolic disorders and immune dysregulation. Metabolomics has emerged as a powerful tool to unravel the immunometabolic mechanisms underlying DKD, facilitating early disease diagnosis, mechanistic pathway interpretation, and therapeutic biomarker discovery. This study aimed to systematically delineate the global research landscape and evolutionary trends of DKD metabolomics, with a key emphasis on immunometabolic interactions.

**Methods:**

Relevant publications on DKD metabolomics published between 2015 and 2025 were comprehensively retrieved from the Web of Science Core Collection and PubMed databases. Multiple bibliometric and visualisation tools, including CiteSpace and the online bibliometric platform (https://bibliometric.com/), were utilised for data processing, visual mapping, and integrated quantitative and qualitative analysis.

**Results:**

A total of 1,410 eligible articles were included in the final analysis, among which 1,110 were sourced from the Web of Science and 299 from PubMed, demonstrating a sustained annual growth in publication volume. China contributed the largest number of publications (663 articles), followed by the United States (207 articles). The University of Michigan and Shandong University were the most productive research institutions. Li Ping and Sharma Kumar were identified as the leading productive and highly cited authors, respectively, with Kidney International recognised as the flagship journal in this research field. Keyword co-occurrence and co-citation analyses confirmed that DKD pathogenesis is predominantly governed by immunometabolic crosstalk. Specifically, renal lipotoxicity, oxidative stress, and insulin resistance trigger persistent renal inflammatory responses, while aberrant glucose metabolism, amino acid dysfunction, and gut microbiota disturbance disrupt renal immune homeostasis via the gut-kidney axis. Branched-chain amino acids and gut microbiota-derived metabolites serve as pivotal immunometabolic biomarkers. Clinical trial data from PubMed further validate the potential applications of these biomarkers, alongside the functional roles of immune regulatory molecules and their correlations with pathological alterations in DKD.

**Conclusions:**

This bibliometric study systematically profiles the global research panorama of DKD metabolomics with a focus on immunometabolic regulation. It consolidates well-established research domains covering lipid- and oxidative stress-induced immune disorders, and further identifies amino acid metabolism-related immunomodulation as a burgeoning research frontier. These findings establish a refined research roadmap for future mechanistic investigations and the development of targeted immunometabolic therapeutic strategies for DKD.

## 1 Introduction

DKD, a severe microvascular complication of diabetes, poses a significant threat to human health (1). Accumulating evidence indicates that immune dysregulation—including systemic low-grade inflammation, gut-kidney axis immune dysfunction, and abnormal activation of inflammatory signaling pathways—is a core driver of DKD pathogenesis, closely intertwined with metabolic disturbances (2, 3). As an emerging field following genomics, transcriptomics, and proteomics, metabolomics offers a powerful approach to decode the crosstalk between immunity and metabolism in DKD. By analysing small-molecule metabolites in biological samples, metabolomics can reveal how metabolic perturbations regulate immune cell function, inflammatory factor release, and barrier immunity, while also identifying immunometabolic biomarkers for early diagnosis and potential therapeutic targets (4, 5). Despite rising recognition of DKD immunometabolism, its research landscape remains fragmented; no comprehensive bibliometric analysis exists, creating an urgent need for such analysis to clarify core questions and emerging frontiers. This study aimed to delineate the research landscape and trends of DKD metabolomics with a specific focus on immunometabolic crosstalk. By analysing publications (2015–2025) retrieved from the Web of Science Core Collection and PubMed databases using CiteSpace and an online bibliometric platform, this study aimed to: 1) identify core research directions of immunometabolism in DKD; 2) summarise key immunometabolic biomarkers and regulatory molecules; 3) highlight emerging frontiers; and 4) provide a roadmap for future research on immunometabolic targets for DKD prevention and treatment.

## 2 Materials and methods

### 2.1 Data sources and search strategy

Literature data were retrieved from the Science Citation Index-Expanded (SCI-Expanded) in the Web of Science Core Collection and the PubMed database. The search query was: TS = (“metabolome” OR “metabolomic profiling” OR “metabolomics” OR “metabolomic” OR “metabonomics” OR “metabonomic” OR “metabolism analysis” OR “metabolic change” OR “metabolic analysis” OR “lipidomics” OR “metabolite levels” OR “lipidomic” OR “metabolic biomarkers” OR “glucose metabolism” OR “lipid metabolism” OR “amino acid metabolism” OR “nucleotide metabolism”) AND TS = (“diabetic nephropathies” OR “nephropathies, diabetic” OR “diabetic nephropathy” OR “diabetic kidney disease” OR “diabetic kidney diseases” OR “kidney disease, diabetic” OR “kidney diseases, diabetic” OR “diabetic glomerulosclerosis” OR “glomerulosclerosis, diabetic” OR “intracapillary glomerulosclerosis” OR “nodular glomerulosclerosis” OR “glomerulosclerosis, nodular” OR “Kimmelstiel-Wilson syndrome” OR “Kimmelstiel Wilson syndrome” OR “syndrome, Kimmelstiel-Wilson” OR “Kimmelstiel-Wilson disease”). The search was limited to publications in English from September 1, 2015, to September 1, 2025. Only “Articles” and “Reviews” were included; other document types, such as Editorials and Meeting Abstracts, were excluded. The PubMed search used the same topics and timeframe. A total of 1410 documents were retrieved.

### 2.2 Data analysis

**Figure 1:**
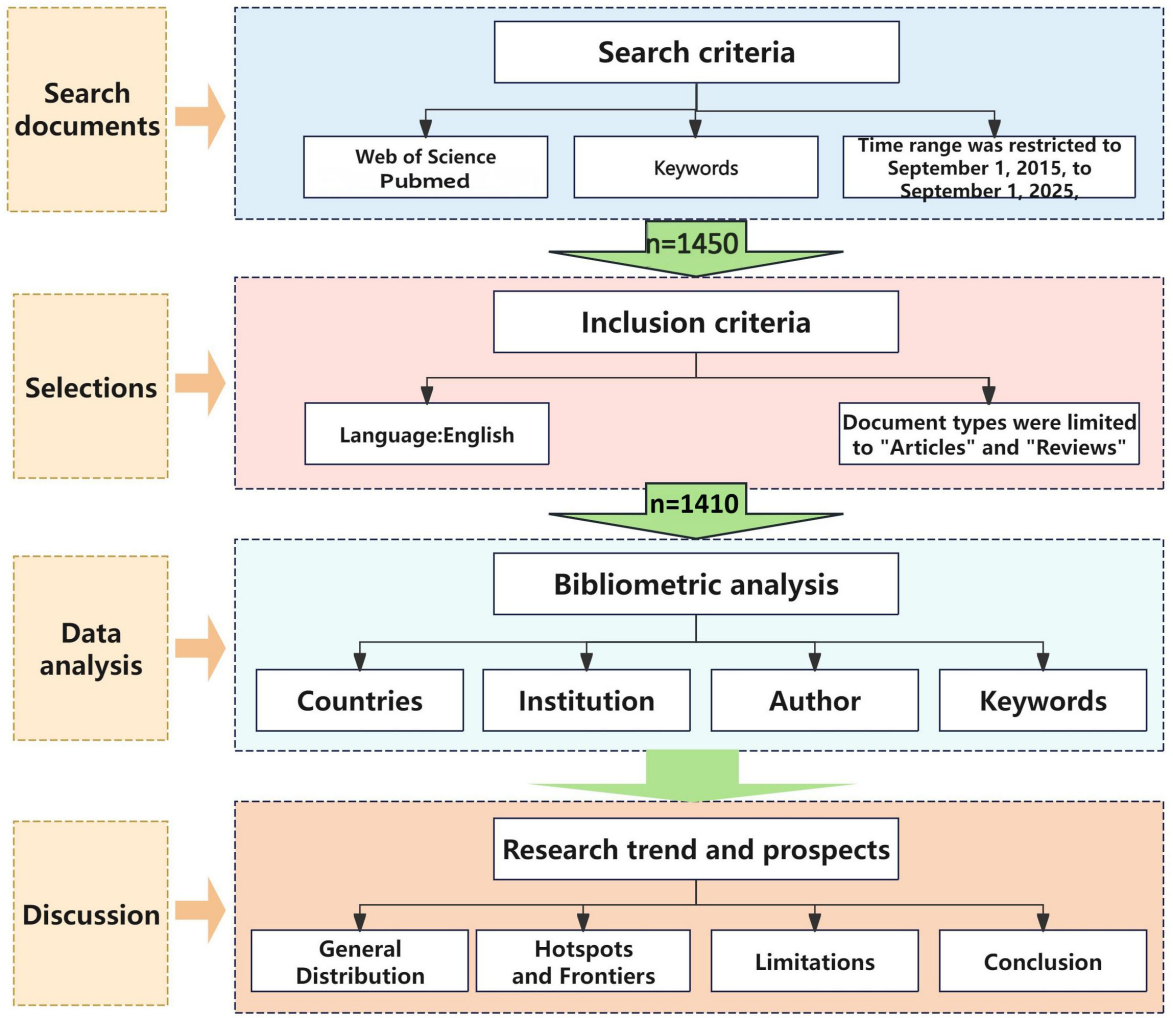
Flowchart of the overall research design. A total of 1450 articles published between 2015–2025 were initially retrieved from Web of Science and PubMed, and 1410 eligible articles were retained after screening based on inclusion criteria. Bibliometric analyses were conducted on countries, institutions, authors, and keywords, followed by a discussion of the field’s publication distribution, research hotspots, limitations, and future prospects.

Two bibliometric tools were used. An online platform (https://bibliometric.com/) was used for national collaboration analysis and publication count comparison. CiteSpace software, developed by Professor Chen, was used to analyse the knowledge structure and distribution (6), specifically for institutional collaboration, author co-citation, and keyword analysis. In the visualisations, nodes represent items (e.g., countries and institutions); their size and colour indicate publication count/frequency and year, respectively, and the connecting lines denote collaborative relationships (7). The retrieval and analysis workflow is illustrated in **Figure 1**.

### 2.3 Duplicate removal workflow

All retrieved publications from the Web of Science and PubMed databases were first imported into EndNote 20 for automatic deduplication using the built-in duplicate detection function. After automated screening, we further performed manual cross-checks based on article titles, first authors, and publication years to remove remaining duplicates, ensuring the uniqueness of all final included literature.

### 2.4 Rationale for the 2015–2025 research window

Over the past decade, immunometabolism has emerged as a rapidly developing interdisciplinary field, and DKD metabolomics research has undergone explosive growth. This ten-year timespan sufficiently captures the latest evolutionary characteristics, prevailing research hotspots, and emerging frontiers in this research area. Publications published before this period generally adopted outdated research frameworks and lacked systematic exploration of immunometabolic mechanisms. Therefore, this time window was selected to guarantee the timeliness and representativeness of the present bibliometric analysis.

### 2.5 Interpretation of CiteSpace visualization parameters

In this study, CiteSpace was employed to conduct bibliometric network analysis. Several key analytical indicators and visual parameters were applied and interpreted as follows. The silhouette score was used to assess the coherence and reliability of cluster classification, with a higher silhouette value indicating better consistency and higher credibility of the clustered research topics. Log-likelihood ratio (LLR) algorithm was adopted to generate automatic cluster labels, which extracts representative thematic terms from titles and abstracts of included literature to objectively reflect the core research focus of each cluster. Betweenness centrality was calculated to identify pivotal nodal items in the co-occurrence network; nodes with high betweenness centrality serve as critical connection hubs linking different research branches and are visually highlighted with purple rings in the network graphs. In addition, a threshold value greater than 0.1 was set for network node screening to filter low-correlation noise relationships, ensuring the reliability and accuracy of the final visualized network structure.

## 3 Results

### 3.1 Bibliometric analysis of countries and institutions

**Figure 2:**
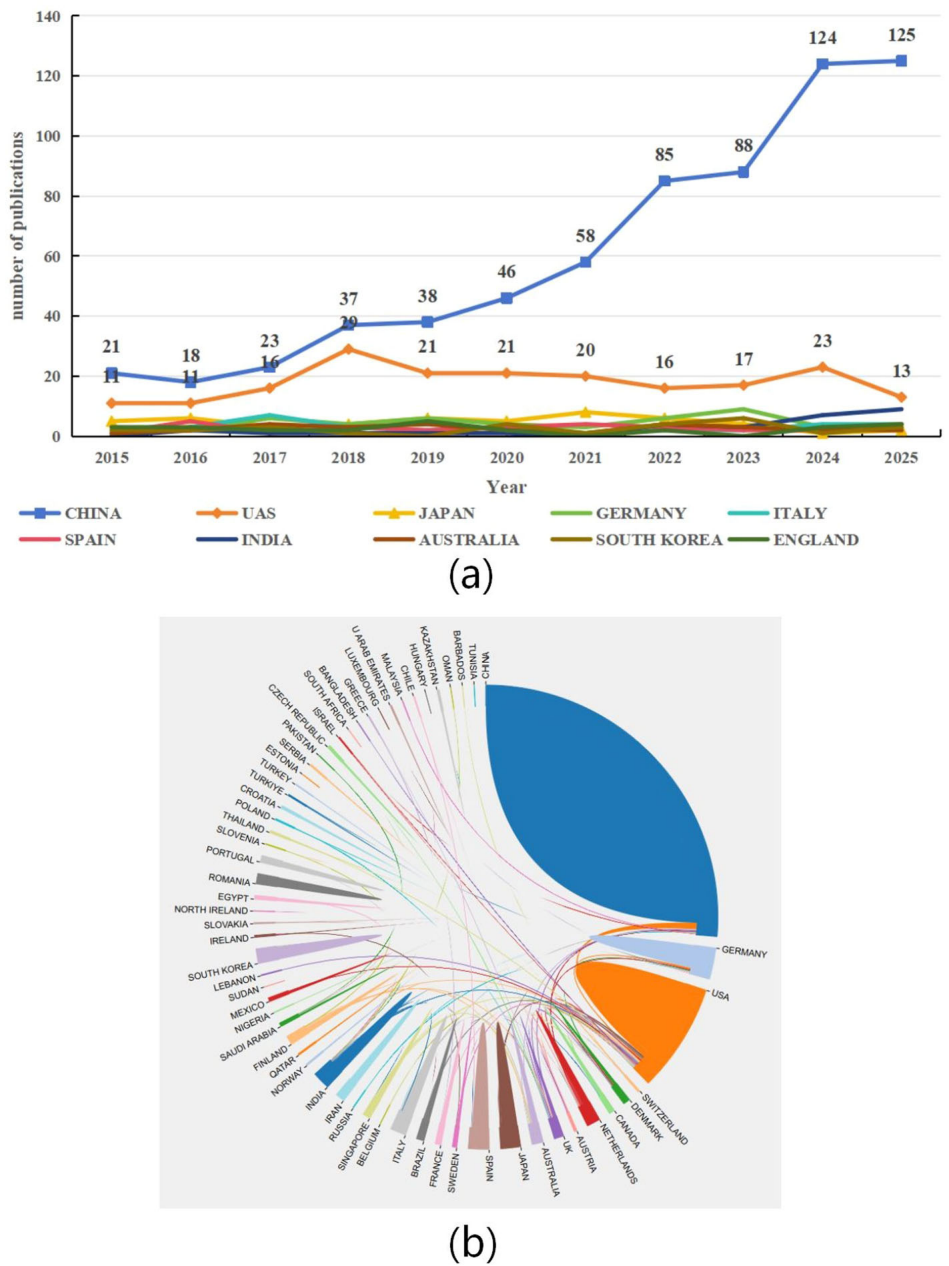
**(a)** Annual publication trends of the top 10 productive countries (2015–2025). **(b)** International academic cooperation network, with sectors representing individual countries and connecting lines indicating cross-national cooperation relationships and intensity.

Based on the annual publication outputs of the world’s top 10 most productive countries from 2015 to 2025, China showed a marked upward trend: starting with 21 publications in 2015, it maintained steady growth in the subsequent years, reaching 124 publications in 2024 and 125 in 2025, with a cumulative total of 663 publications over the decade (**Figure 2a**). The United States exhibited relative stability with minor fluctuations, starting with 15 publications in 2015 and declining to 13 by 2025, with a total of 207 publications. Japan showed a gradual decline, with the number of publications decreasing from approximately eight in 2015 to approximately five in 2025. In contrast, Germany, Italy, Spain, India, Australia, South Korea, and the United Kingdom maintained relatively low and stable publication volumes throughout this period.

Cooperative relationships among countries are illustrated in **Figure 2b**, where nodes represent countries and connecting lines indicate collaborative links. Node size reflects a country’s centrality within the cooperative network. The large blue and orange nodes, representing major countries such as China and the United States, account for a substantial proportion, underscoring their pivotal roles in international collaboration. The density and distribution of the coloured lines further reflect the intensity and scope of these cooperative efforts.

Among the top nine institutions by publication volume, the University of Michigan yielded the highest total number of publications (n = 50), followed by Shandong University (n = 37), the University of Miami (n = 34), and the University of California, San Diego (n = 29). Among these institutions, five are from the United States, two from China, and one each from Sweden, Finland, and Japan (**Table 1**).

**Table 1:** Top nine institutions by number of publications.

Institution name	Total number of articles	Total citations	Average citations
Univ Michigan	50	696	13.92
Univ Miami	34	481	14.15
Univ Calif San Diego	29	462	15.93
Shandong Univ	37	274	7.41
Karolinska Inst	6	210	35.00
Huazhong Univ Sci & Technol	37	208	5.62
Univ Helsinki	25	201	8.04
NIDDK	10	201	20.10
Tohoku Univ	12	200	16.67

This table lists the top 9 institutions with the highest publication output in this research field, sorted by the total number of published articles. Average citations were adopted to evaluate the citation popularity and academic recognition of each institution’s research achievements.

Univ, University; Calif, California; Inst, Institute; Sci & Technol, Science and Technology; NIDDK, National Institute of Diabetes and Digestive and Kidney Diseases.

**Figure 3:**
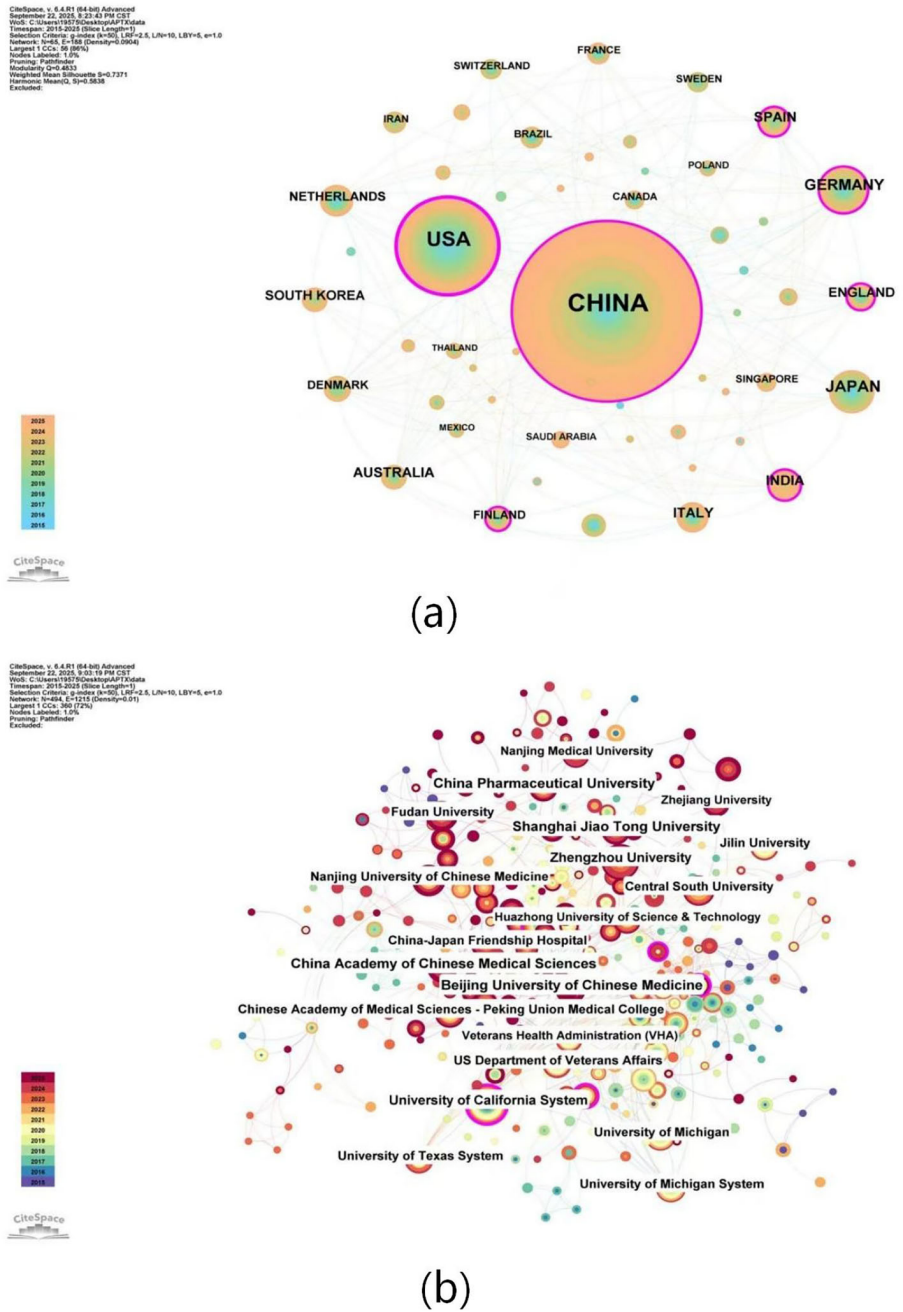
**(a)** National collaboration network. Node size denotes publication volume, line thickness represents cooperation frequency, and node color indicates the earliest publication year of each country. **(b)** Institutional collaboration network, where node size and links reflect institutional publication output and global academic partnerships, respectively.

The country/region map generated by CiteSpace uses the node size to represent publication frequency. China has the largest node, indicating the highest output, followed by the United States. The purple outer ring on each node indicates its centrality, signifying its importance in the research network. Countries with notable centralities include the United States, Japan, and Germany (**Figure 3a**).

Beijing University of Chinese Medicine led the institutional publication output, followed by China Academy of Chinese Medical Sciences, Nanjing University of Chinese Medicine, China Pharmaceutical University, and Fudan University. Chinese institutions such as Shanghai Jiao Tong University and Zhejiang University form a relatively dense collaborative cluster. However, collaborative links between these Chinese institutions and those from other regions, such as the University of California and the University of Michigan in the United States, appear to be relatively limited (**Figure 3b**).

### 3.2 Bibliometric analysis of authors and co-authors

**Figure 4:**
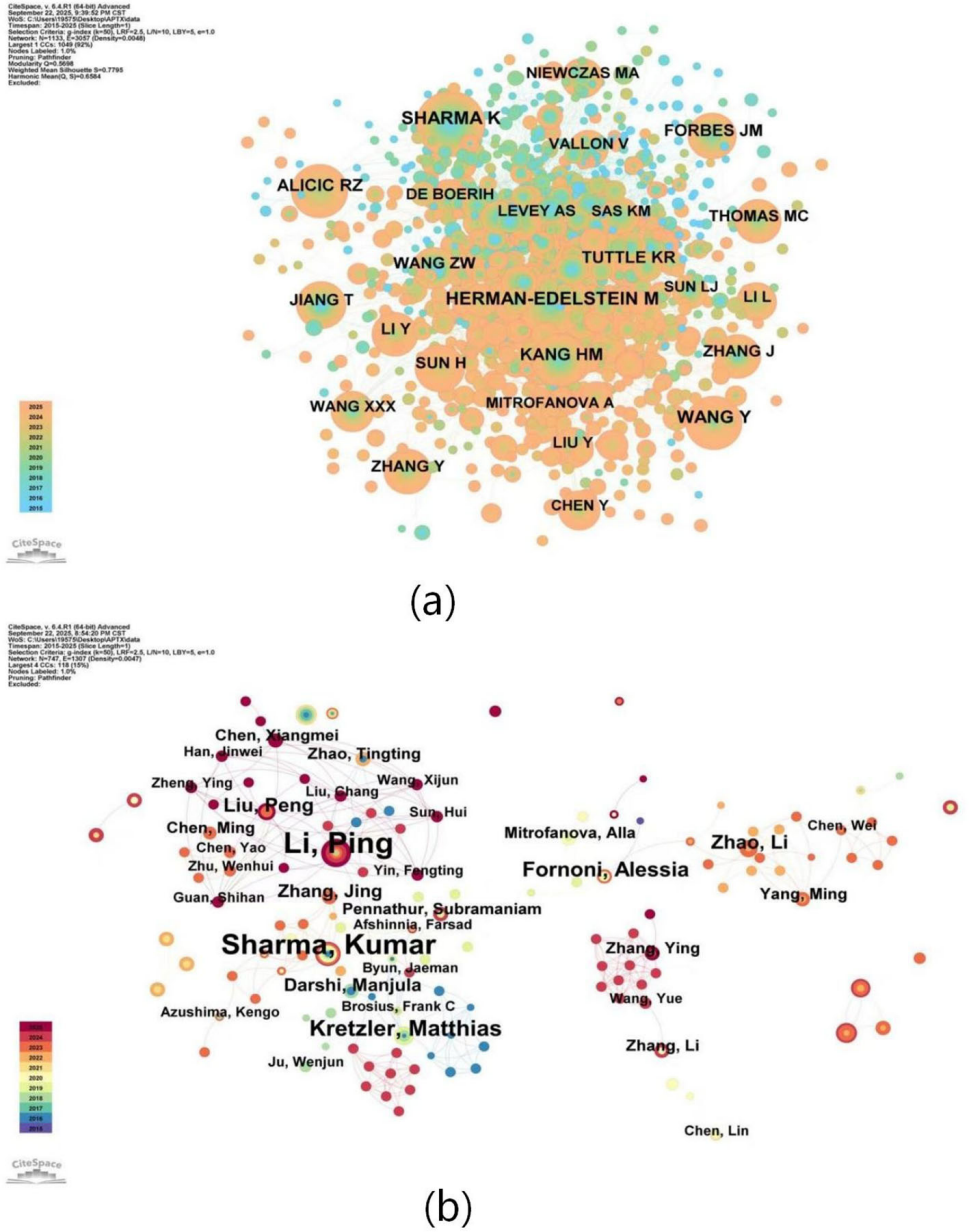
**(a)** Author collaboration network. Node size correlates with individual publication output, node color represents the earliest publication year, and lines indicate author collaborations. **(b)** Author co-citation clustering network. Node size shows cumulative co-citation frequency, color clusters denote closely connected research teams, and lines represent co-citation associations.

Several authors contributed to multiple publications in this study. The five most prolific authors were Li Ping (20 articles), Sharma Kumar (16 articles), Levi Moshe (nine articles), Liu Zhangsuo (nine articles), and Liu Dongwei (eight articles). Li Ping was a major contributor, as detailed in **Table 2** and visualised in **Figure 4a**, which displays author distribution and collaboration.

**Table 2:** Top 15 most productive authors.

Rank	Count	Author
1	20	Li, Ping
2	16	Sharma, Kumar
3	9	Levi, Moshe
4	9	Liu, Zhangsuo
5	8	Liu, Dongwei
6	8	Kretzler, Matthias
7	7	Fornoni, Alessia
8	6	Liu, Peng
9	6	Zhou, Yang
10	6	Zhao, Li
11	5	Pan, Shaokang
12	5	Darshi, Manjula
13	5	Zhang, Jing
14	5	Zhang, Yan
15	5	Rossing, Peter

This table lists the top 15 authors with the highest publication output in the research field.

Co-citation analysis helps identify influential, highly cited scholars. Using the g-index (k = 50) in CiteSpace, the node size represents the citation frequency, with larger nodes indicating higher citation frequencies. The links between the nodes depict co-citation relationships. Sharma Kumar was the most frequently cited author, followed by Li Ping, Kretzler Matthias, and Darshi Manjula. Node characteristics reflect the influence of scholars in this field (**Figure 4b**).

### 3.3 Bibliometric analysis of co-cited journals

The leading journals based on citation metrics are Kidney International (17 articles, 125 total citations, 7.35 average citations), Journal of the American Society of Nephrology (7 articles, 116 total citations, 16.57 average citations), and Nature Medicine. Centrality measures a journal’s importance within the citation network, based on how often a node appears on the shortest path. Among these, Cell Metabolism has a relatively high average citation score (32.50), indicating its significant influence in this field (**Table 3**).

**Table 3:** Journal impact.

Journal name	Total number of articles	Total citations	Average citations
Kidney International	17	125	7.35
Journal Of The American Society Of Nephrology	7	116	16.57
Nature Medicine	1	115	115.00
International Journal Of Molecular Sciences	27	91	3.37
Scientific Reports	22	90	4.09
Jci Insight	6	90	15.00
Nature Reviews Nephrology	9	89	9.89
Frontiers In Endocrinology	36	86	2.39
Cell Metabolism	2	65	32.50
Diabetologia	10	64	6.40

This table summarizes the top journals sorted by total citations of included papers.

JCI, Journal of Clinical Investigation.

### 3.4 Bibliometric analysis of co-cited references

**Figure 5:**
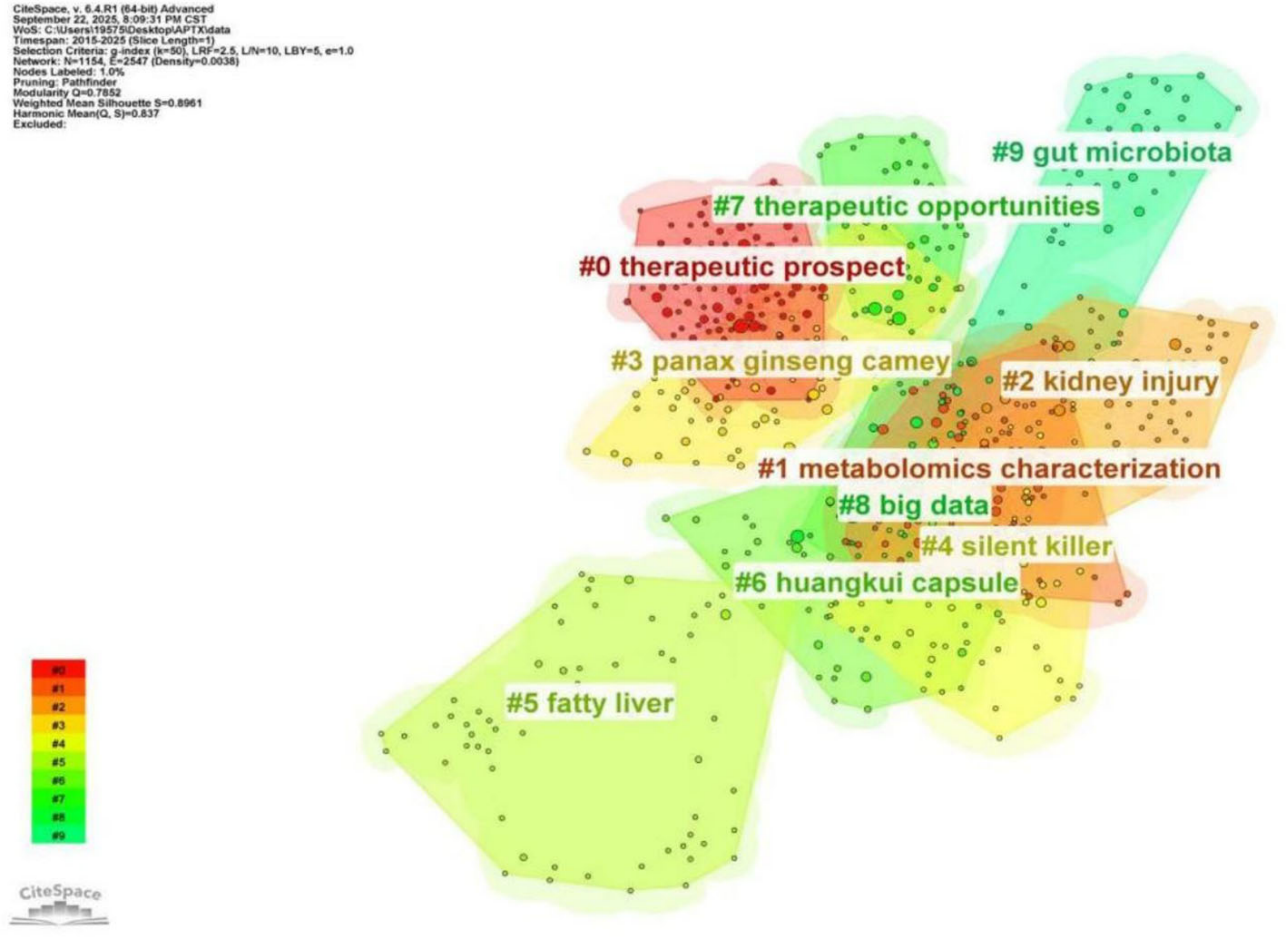
Clustering map of co-cited literature. Dots represent individual references, and colored regions indicate 10 validated thematic research clusters with favorable modularity and silhouette values.

Frequent co-citation clustering identified topics including ‘metabolomics characterization’, ‘kidney injury’, ‘fatty liver’, ‘gut microbiota’, and ‘therapeutic opportunities’. The ‘metabolomics characterization’ cluster was particularly prominent. Burst detection indicated notable short-term attention to topics such as ‘big data’. The main thematic clusters identified were ‘therapeutic prospect’, ‘metabolomics characterization’, ‘kidney injury’, ‘panax ginseng camey’, and ‘silent killer’ (**Figure 5**).

### 3.5 Bibliometric analysis of keywords

**Figure 6:**
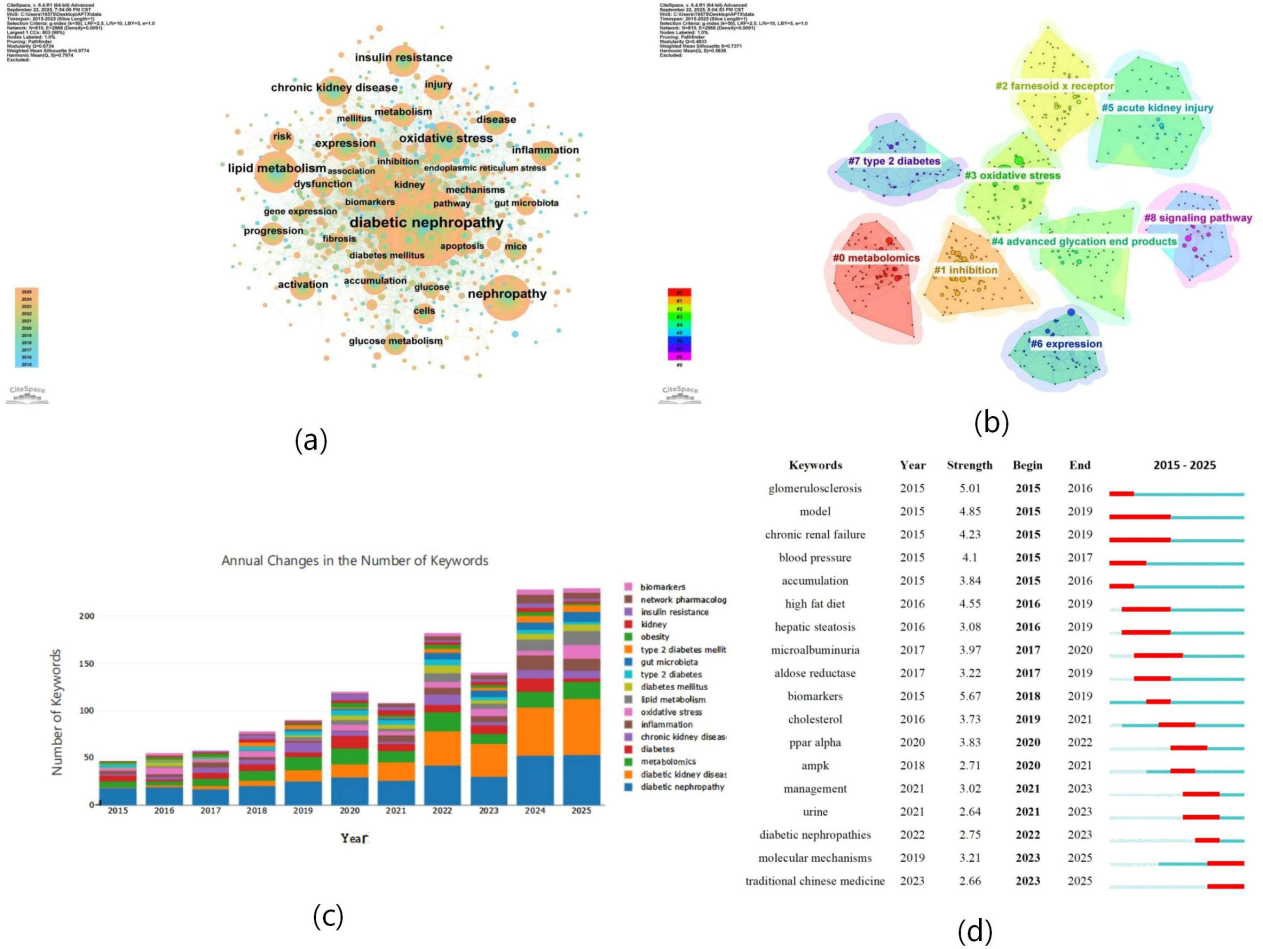
**(a, b)** Keyword co-occurrence network and clustering distribution, illustrating keyword frequency, emergence time, co-occurrence relationships, and core research themes. **(c)** Annual variation in the number of clustered keywords (2015–2025). **(d)** Top 18 keywords with strong citation bursts, showing burst intensity and active time intervals.

Keyword node analysis identified the most frequently used terms as ‘diabetic nephropathy’, ‘nephropathy’, ‘lipid metabolism’, and ‘oxidative stress’ (**Figure 6a**). Keyword clustering revealed distinct groups, prominently featuring ‘metabolomics’, ‘oxidative stress’, ‘advanced glycation end products’, and ‘signaling pathway’ (**Figure 6b**; **Table 4**). **Figure 6c** illustrates the annual frequency of keywords from 2015 to 2025, showing a clear upward trend from a low in 2015 to a peak in 2024–2025.

**Table 4:** The cluster information of keywords.

ClusterID	Size	Silhouette	Year	LLR
0	96	0.63	2019	metabolomics; mass spectrometry; biomarkers; amino acids; targeted metabolomics
1	85	0.739	2019	inhibition; inflammation; lipid metabolism; network pharmacology; fibrosis
2	74	0.756	2019	farnesoid x receptor; fatty acid oxidation; bile acids; treatment; glomerulosclerosis
3	60	0.742	2018	oxidative stress; diabetic kidney disease; diabetic nephropathy; diabetes; glycogen synthase
4	58	0.798	2018	advanced glycation end products; astaxanthin; growth factor; diabetic kidney injury; renal fibrosis
5	56	0.631	2020	acute kidney injury; kidney transplantation; post-transplant diabetes mellitus; mitochondrial fission; chronic kidney disease
6	55	0.805	2020	expression; pathway; activation; metabolism; amino acid metabolism
7	52	0.797	2018	type 2 diabetes; diabetic nephropathies; sclerostin; outcome; diabetes mellitus
8	51	0.773	2018	signaling pathway; glucose metabolism; metabolomics; inflammation; high-fat diet

A total of 9 keyword clusters were obtained in this study. The silhouette coefficient of all clusters ranges from 0.63 to 0.805, all greater than 0.6, indicating that the clustering result is reliable and reasonable.

LLR, Log-Likelihood Ratio.

The keyword burst detection spectrum reveals that the term ‘biomarkers’ exhibited relatively high burst strength, whereas keywords including ‘model’ and ‘chronic renal failure’ sustained their prominence over consecutive years. At present, ‘molecular mechanisms’ and ‘traditional Chinese medicine’ have been identified as emerging research hotspots, with the latter garnering substantial attention since 2023 (**Figure 6d**).

### 3.6 Clinical practices of metabolomics in diabetic nephropathy

**Figure 7:**
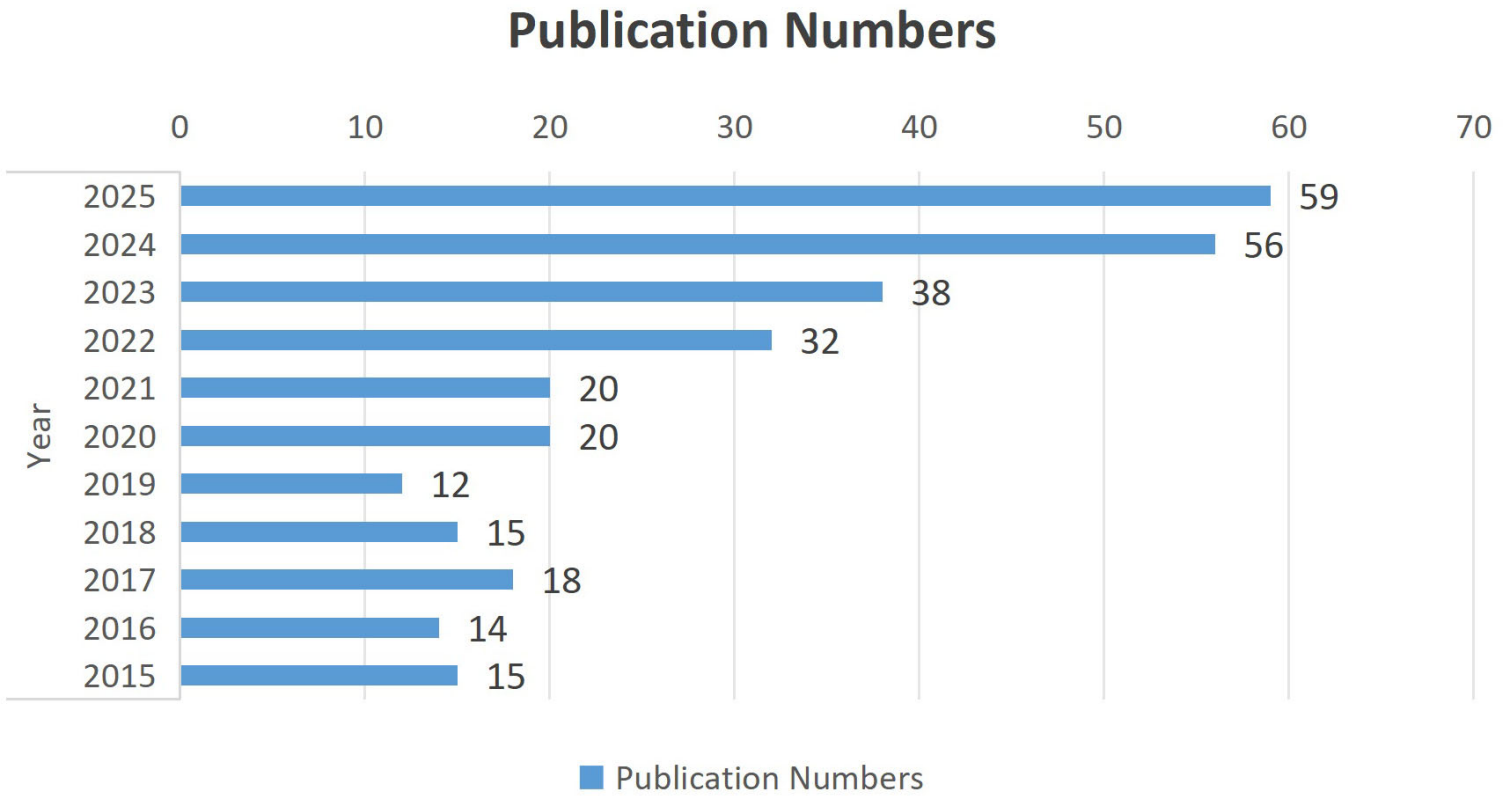
Annual publication volume of metabolomics research on diabetic nephropathy (2015–2025). The chart displays yearly publication counts, with horizontal bars and attached numbers quantifying annual research output.

To further investigate the current status of clinical applications pertaining to metabolomics in DKD, we conducted a literature search on the same topic and within the identical timeframe in the PubMed database (**Figure 7**). Based on the search outcomes, we classified the research categories of metabolomics in DKD into five major types: Serum Metabolomics, Plasma Metabolomics, Tissue Metabolomics, Cellular Metabolomics, and Urine Metabolomics (**Table 5**). Key findings are summarised as follows. Regarding critical metabolites, the identified ones include serum phosphatidylcholine, fatty acids (particularly ω-3 long-chain fatty acids) (8), glycerol-3-galactoside, 1,5-anhydroglucitol, norvaline, L-aspartic acid (9), a set of 18 urinary metabolites (10), and urinary tricarboxylic acid cycle organic anions (11). In terms of key molecules, α2-macroglobulin, cathepsin D, and CD324 are involved as core regulatory factors (12). For relevant pathways, the research focuses on energy metabolism, mitochondrial function, and endothelial function pathways (13). With respect to pathological changes, these metabolic and molecular alterations are associated with mitochondrial dysfunction (14), adaptive/maladaptive tubular subtypes, glomerular basement membrane thickening (15), and tubular injury (16).

**Table 5:** Clinical trials related to metabolomics of diabetic nephropathy.

Category	Key findings	Title	Journal	Year
Serum Metabolomics	Serum phosphatidylcholine; fatty acid; ω-3 long-chain fatty acids	Serum levels of plasmalogens and fatty acid metabolites associate with retinal microangiopathy in participants from the finnish diabetes prevention study	Nutrients	2021
	Glycerol-3-galactoside; α2-macroglobulin/cathepsin D/CD324	Serum integrative omics reveals the landscape of human diabetic kidney disease	Mol Metab	2021
	Energy/mitochondrial/endothelial function pathways	A metabolomics-based molecular pathway analysis of how the sodium-glucose co-transporter-2 inhibitor dapagliflozin may slow kidney function decline in patients with diabetes	Diabetes Obes Metab	2020
Plasma Metabolomics	1,5-anhydroglucitol/norvaline/L-aspartic acid correlate	1,5-Anhydroglucitol predicts CKD progression in macroalbuminuric diabetic kidney disease: results from non-targeted metabolomics	Metabolomics	2018
Tissue Metabolomics	Adaptive/maladaptive tubular subtypes and GBM thickening	Attenuated kidney oxidative metabolism in young adults with type 1 diabetes	J Clin Invest	2024
Cellular Metabolomics	Tubular injury	The mitochondria-targeted metabolic tubular injury in diabetic kidney disease	Cell Physiol Biochem	2019
Urine Metabolomics	A set of 18 urinary metabolites	Baseline urinary metabolites predict albuminuria response to spironolactone in type 2 diabetes	Transl Res	2020
	Urine tricarboxylic acid (TCA) cycle organic anions (OAs)	Urine tricarboxylic acid cycle signatures of early-stage diabetic kidney disease	Metabolomics	2021
	Mitochondrial dysfunction	The effects of atrasentan on urinary metabolites in patients with type 2 diabetes and nephropathy	Diabetes Obes Metab	2017

This table systematically sorts out clinical trials of diabetic nephropathy metabolomics published from 2015 to 2025, which are classified into 5 categories according to sample sources: serum, plasma, kidney tissue, cell and urine metabolomics.

CKD, Chronic Kidney Disease; GBM, Glomerular Basement Membrane; TCA, Tricarboxylic Acid; OAs, Organic Anions.

## 4 Discussion

A comprehensive bibliometric analysis of DKD metabolomics research spanning the past decade was implemented using CiteSpace in this study, with the specific objective of decoding the immunometabolic crosstalk that underpins DKD pathogenesis. Systematic data collection and visualisation enabled the identification of principal research orientations, influential scholars, and emerging frontiers in DKD immunometabolism, yielding critical insights to inform subsequent immunology and metabolomics investigations.

### 4.1 Publication volume trends

Over the past decade, the field of DKD metabolomics has experienced significant and consistent growth in publications, reflecting a steadily increasing research interest. Technological advancements are a primary driver, and the widespread application of mass spectrometry and nuclear magnetic resonance in metabolomics has enabled the more precise detection and analysis of metabolites in biological samples, providing rich and accurate data for DKD research. Among the prolific authors, scholars such as Li Ping and Sharma Kumar have achieved notable results. Li Ping, with 20 relevant publications, focused on key areas, including the application of metabolomic techniques to identify biomarkers for early diagnosis and the investigation of the role of specific metabolites in pathogenesis, providing both theoretical and practical support for the advancement of the field. Core journals such as Kidney International and Journal of the American Society of Nephrology have played vital roles in advancing this research domain.

### 4.2 Distribution of research institutions and countries

Significant disparities in research output were observed at the national level between 2015 and 2025. China demonstrated a remarkable upward trend, with annual publications increasing from 21 in 2015 to 125 in 2025, totaling 663 publications over the last decade. The United States maintained a relatively stable output with minor fluctuations, declining to 13 publications in 2025, totaling 207 publications over the 10-year period. Japan experienced a gradual decline, with approximately five articles being published in 2025. Seven other countries, including Germany and Italy, maintained low but stable publication volumes.

At the institutional level, University of Michigan in the United States ranked first, with 50 publications, followed by University of Miami (34 publications) and University of California, San Diego (29 publications). Shandong University, China, also demonstrated exceptional performance, with 37 publications. Other notable institutions include universities in Sweden, Finland, and Japan, as well as Chinese institutions such as Beijing University of Chinese Medicine and China Academy of Chinese Medical Sciences. Collaborative clusters have formed among institutions such as Shanghai Jiao Tong University and Zhejiang University. However, cooperation between Chinese universities and certain U.S. institutions remains limited, highlighting the need to strengthen international research collaboration.

### 4.3 Lipid metabolism, oxidative stress, and insulin resistance as immunometabolic nodes

Keyword co-occurrence and co-citation clustering analyses in this bibliometric study confirm that lipid metabolism, oxidative stress, and insulin resistance (IR) are the three core immunometabolic nodes dominating current DKD metabolomics research, which critically bridge renal metabolic disorders and immune dysregulation.

Dysregulated lipid metabolism acts as a pivotal initiator of renal immune activation in DKD. Ectopic renal lipid accumulation triggers lipotoxicity and oxidative stress, forming a self-amplifying lipid-immune vicious cycle and accelerating renal fibrosis (17, 18). Targeted lipid-modulating agents including statins, fenofibrate, and dapagliflozin exert renoprotective effects via dual regulation of metabolic homeostasis and immune responses (19–21), verifying lipid metabolism as a core therapeutic target of DKD immunometabolism.

As a central intermediate node, oxidative stress connects DKD metabolic abnormalities and immune dysfunction. Excessive ROS disrupts immune homeostasis, activates NF-κB signaling, and induces inflammatory cascade reactions, driving sustained renal pathological damage (22–24). Additionally, IR exacerbates DKD progression by synergizing metabolic disorders and immune inflammation, while IR-related lipid and hemodynamic abnormalities further trigger renal immune activation and proteinuria (25, 26).

Collectively, this bibliometric analysis systematically clarifies the core status of the three immunometabolic nodes in DKD research, providing targeted and novel directions for precise immunometabolic therapy of DKD.

### 4.4 Co-citation clustering analysis of references

Our co-citation clustering analysis delineates two prominent emerging research clusters in DKD metabolomics. The first cluster focuses on multi-dimensional metabolomic applications in DKD. Metabolomic profiling enables early auxiliary diagnosis and prognostic evaluation of DKD via screening differential blood and urinary metabolites and disease-specific proteins (27, 28). It also clarifies aberrant metabolic pathways underlying DKD pathogenesis and facilitates the exploration of novel therapeutic targets such as the SIRT2/HIF-1α/VEGFA axis, providing metabolic-level evidence for DKD clinical intervention and drug development (29–32).

The second core cluster highlights the gut-kidney axis as a frontier research direction. Bibliometric results confirm that gut microbiota (GM) dysbiosis mediates immunometabolic crosstalk to drive DKD progression by disrupting intestinal barrier and triggering persistent renal inflammatory responses (33–35). Gut microbiota-derived functional metabolites exert renal protective effects, while traditional Chinese medicine can regulate GM composition to remodel intestinal and immune homeostasis, offering innovative targeted strategies for DKD immunometabolic therapy (36–39).

### 4.5 Keyword salience analysis in metabolomics studies of diabetic nephropathy

Our keyword salience analysis of DKD metabolomics studies identifies two pivotal research hotspots: glucose metabolism-hemodynamics crosstalk and amino acid metabolic dysregulation. The first core theme highlights the central pathogenic interaction between glucose metabolic disorders and renal hemodynamic abnormalities in DKD. Key molecules including GLUT1, TGF-β1, and VEGF mediate a persistent pathogenic cycle coupling hyperglycemia and hemodynamic stress, which triggers podocyte damage, glomerulosclerosis, and irreversible renal deterioration (40, 41).

Amino acid metabolism dysregulation constitutes another vital research node closely linked to DKD immunometabolic disorders. Aberrant metabolism of BCAAs, taurine and other key amino acids modulates renal oxidative stress and immune inflammation, serving as reliable biomarkers for DKD progression (42–45). Targeted regulation of amino acid metabolic homeostasis has been verified to ameliorate renal pathological injuries, representing a promising immunometabolic therapeutic direction for DKD (46).

### 4.6 Limitations

This study conducted a comprehensive bibliometric analysis of DKD-related metabolomics studies published between 2015 and 2025. However, several notable limitations should be addressed. First, the literature retrieval relied solely on mainstream academic databases with unified inclusion criteria, which may omit relevant studies from non-core databases, regional repositories or newly developed platforms, potentially undermining the comprehensiveness of the analysis. Second, only English peer-reviewed articles were included in this study. The exclusion of non-English publications may introduce geographical and publication bias and fail to fully represent the global research landscape of DKD metabolomics. Third, all bibliometric results and visualisations were generated using specialised analytical software. The inherent algorithmic mechanisms and standardised parameter settings of such software may produce analytical deviations that affect the accuracy of hotspot recognition, co-occurrence mapping and research trend prediction. Finally, this study did not fully elaborate practical translational challenges in DKD metabolomics research, including technical obstacles in multi-omics data integration, uncertainties in clinical biomarker validation, and methodological and resource limitations of high-quality TCM clinical trials. Collectively, these limitations suggest that future studies should adopt more diverse database sources, multi-language inclusion strategies, and optimised bibliometric workflows to overcome existing deficiencies and advance the clinical translation of DKD metabolomics research.

## 5 Conclusion

This study systematically analyzed the global research landscape of DKD metabolomics from 2015 to 2025, with a specific focus on immunometabolic crosstalk, using comprehensive bibliometric approaches. The results revealed a steadily expanding body of literature, with China and the United States (USA) as the leading contributing countries, and the University of Michigan and Shandong University as prominent research institutions. Our findings highlight that DKD pathogenesis is driven by complex immunometabolic interactions: ectopic renal lipid accumulation and ACACB variants induce lipotoxicity and activate renal immune cells; excessive reactive oxygen species (ROS) trigger oxidative stress-mediated inflammatory signaling; insulin resistance drives metabolic perturbations and inflammatory pathway activation; and dysregulated glucose, amino acid, and gut microbiota metabolism disrupt immune homeostasis via the gut–kidney axis and interconnected immunometabolic networks. Branched-chain amino acids (BCAAs), gut–kidney axis metabolites, and inflammatory signaling molecules emerge as key immunometabolic biomarkers and potential therapeutic targets.

Beyond branched-chain amino acids, asprosin has emerged as a novel immunometabolic adipokine biomarker that is strongly associated with the progression of DKD. Circulating asprosin levels are correlated with renal injury under diabetic conditions, and this relationship is potentially mediated by inflammatory responses (47). Clinically, elevated plasma asprosin concentrations have been observed in patients with type 2 diabetes mellitus (T2DM) at both the pre-DKD and early DKD stages, and such alterations are closely correlated with key indicators reflecting DKD severity (48). As a newly identified immunometabolic regulator, asprosin warrants further clinical validation for its potential utility as an early diagnostic biomarker and therapeutic target for DKD.

Based on our bibliometric analysis, future immunometabolic research in DKD should focus on three core areas: (1) elucidating the precise mechanisms by which gut microbiota-derived metabolites regulate renal immune cell subsets via the gut–kidney axis; (2) validating immunometabolic biomarkers for clinical application in predicting DKD progression and responsiveness to immunomodulatory therapies; and (3) developing targeted immunometabolic interventions that simultaneously correct metabolic disturbances and restore immune homeostasis. Furthermore, multi-omics integration, including metabolomics, immunomics, and transcriptomics, is essential for mapping the global immunometabolic regulatory network in DKD, and large-scale clinical trials are warranted to validate the efficacy of these immunomodulatory strategies.

## Data Availability

The raw data supporting the conclusions of this article will be made available by the authors, without undue reservation.
